# Modification of surface characteristics of ophthalmic biomaterial-polymethyl methacrylate induced by cobalt 60 gamma irradiation

**DOI:** 10.1371/journal.pone.0291344

**Published:** 2023-09-15

**Authors:** Dong Qin, Juan Guo, Ming Liang, Ling Qin, Ling Chen, Weimin He

**Affiliations:** 1 Ophthalmology Department, The Third People’s Hospital of Chengdu, Chengdu, P R China; 2 Department of Ophthalmology, West China Hospital, Sichuan University, Chengdu, P R China; Universidad Tecnica de Ambato, ECUADOR

## Abstract

This study aims to observe the accelerated aging effect of ^60^Co gamma (γ) irradiation on poly (methyl methacrylate) (PMMA) under extreme conditions and determine the influence of different media states on aging. PMMA samples were prepared at room temperature under varying media conditions, including air and deionized water immersion. Then, the samples were irradiated with different doses (50, 250, 500, and 1000 KGy) of ^60^Co γ-rays. The compositional changes of the PMMA samples exposed to the rays at different periods were determined via Fourier transform infrared spectroscopy. The light transmission of the samples was characterized through ultraviolet–visible spectrophotometry, and the surface wettability of the samples was assessed via water contact angle measurements. Surface and microscopic changes in material morphology were analyzed using optical microscopy, ImageJ software, and scanning electron microscopy. Relative molecular mass and glass transition temperature were analyzed via gel permeation chromatography and differential scanning calorimetry. Thus, a comprehensive analysis of the effect of ^60^Co γ irradiation on the aging properties of PMMA was performed.

## 1 Introduction

Biomaterials have been broadly used in the field of ophthalmology. Each application has different requirements. In general, an ideal ophthalmic material is required to have stable physicochemical properties, good biocompatibility, no antigenicity, no rejection, good tissue tolerance, no irritation, and no uncomfortable feeling when implanted in the eye; it should also have its own suitable properties for different applications [1]. In the past 20 years, with the innovations in science and technology and the rapid progress of ophthalmology, people have increasing requirements for ophthalmic materials and require better functional ophthalmic biomaterials to be used in various fields of ophthalmology [2]. Among the earliest and most widely applied ophthalmic biomaterials, poly (methyl methacrylate) (PMMA) is used in ophthalmology, including external and internal eye, and ophthalmic medical devices, such as protective lenses, spectacles, keratoplasty lenses, contact lenses, artificial corneas, artificial lenses, stents, and prosthetic materials. PMMA is known for its unique optical and mechanical properties, such as high transparency and brightness, strong heat resistance, hardness, aesthetic quality, low cost, and easy handling. PMMA has been the most suitable and popular biomaterial in ophthalmology [3].

With the increasing development of ophthalmic biomaterials, PMMA has been used in a growing number of application scenarios. In environments with high altitude, radiation exposure, and extreme temperatures, the safety and effectiveness of a material are the primary safety factors that must be considered, and the failure of a material may threaten the lives of people. Evaluating the ability of polymer materials to resist radiation and aging is crucial for selecting materials to be used in extreme and complex environments. For example, to ensure proper aircraft flight and air safety, aerospace clear glass components must have excellent optical properties, sufficient toughness, and long service life. However, long-term high-intensity exposure to light, high temperature, and humidity can easily lead to color changes, loss of light transmission, and degradation of mechanical properties [4]. Therefore, studying the effects of aging due to environmental factors on the performance of aerospace lenses is important. Researchers worldwide have investigated the performance evolution patterns and mechanisms of aerospace Perspex™ in different environments by simulating environmental parameters, such as atmosphere, thermal oxidation, humidity, and ultraviolet (UV) light. They have concluded that sunlight is the major factor that contributes to the variation of PMMA properties [5, 6].

Radiation may degrade the mechanical properties of a material so that it is no longer mechanically suitable. Materials are exposed to a variety of radiation sources, including X-rays, gamma rays and neutrons. Polymeric materials are increasingly used for technological applications under irradiated environmental conditions, and the ability to predict the lifetime of a material while it is irradiated remains a limiting factor for many existing radiation technologies. Although the exposure of biological materials to large doses of nuclear radiation is less, there is no doubt that the possible deterioration by radiation could lead to serious consequences. Polymers may exhibit a wide range of radiation effects; the formation or breaking of chemical bonds often leads to irreversible effects that alter the chemical, thermal or mechanical properties of the material. The reactions that occur in irradiated polymers can be broadly categorized into two types, cross-linking and chain breaking. Generally, cross-linking increases the strength of the polymer material, while the opposite happens in case of chain breaking. However, the degree and direction of change is extremely sensitive to the material. Many scholars have discovered new materials by studying the effects of various parameters on optical properties, such as concentration [7], wavelength [8], incident intensity [9], temperature [10], salt concentration [11], glucose concentration [12], and pH [13]. The development of radiation technology in the last 15 years, such as the use of gamma radiation technology, has led to the intensive study and improvement of various properties of some materials. Macknowski et al. [14] investigated the effect of gamma (γ) rays on the mechanical properties and susceptibility of materials. Rani et al. [15] examined the effect of γ-rays on the structural and thermal properties of hydroxyl compounds. Mirzayev et al. [16–18] investigated the correlation between irradiation dose and surface properties of compounds from various aspects, such as the formation of color centers and the concentration of defects in boron carbide, radiation absorption dose of silicide hexoboride and the thermally stable temperature range for unirradiated and irradiated compound. However, the irradiation doses used in these studies were extremely limited. Moreover, no research has yet simulated changes in materials under extreme γ-ray irradiation because few facilities can irradiate a sufficiently large area with uniform doses to allow reasonable measurements of mechanical properties. Meanwhile, few reports have been made on the property changes of PMMA materials under high-intensity γ-ray irradiation in China due to its relatively high cost. As an irradiation source, ^60^Co γ exhibits the benefits of easy preparation, moderate half-life, strong penetration of irradiated γ-rays, and high energy; it is commonly used in the sterilization of food and drugs in daily life. It is a suitable accelerated test method for simulating the radiation resistance of polymer materials [19, 20].

In the current work, the surface morphology, physicochemical properties, and optical properties of PMMA in different media were investigated after accelerated aging following exposure to irradiation. The changes in the surface morphology and physicochemical properties of PMMA in different media after irradiation were investigated using Fourier transform infrared (IR) spectroscopy (FTIR), gel permeation chromatography (GPC), and differential scanning calorimetry (DSC). The effects of γ-rays on the surface morphology and optical properties of PMMA were investigated using scanning electron microscopy (SEM), optical microscopy, ImageJ software, and spectroscopic measurements. Changes in the surface energy of PMMA before and after irradiation were calculated and analyzed by studying the changes in hydrophobicity of PMMA after irradiation. The aging of PMMA materials under extreme irradiation is reported to lay the foundation for the subsequent development of PMMA coating research. Accelerated aging by using γ-ray, which can simulate an environment with sunlight, humidity, and heat, is a suitable accelerated test method for simulating the weathering of polymer materials. Given its relatively high cost, only a few domestic reports have been made on the performance changes of polymer materials, particularly ophthalmic biomaterials, when exposed to high-intensity irradiation. Accordingly, the morphology, structure, and properties of PMMA under different media were observed, characterized, and tested in the current study by conducting Co-γ test with a maximum dose of 1000 kGy on aerospace lenses to explore the behavioral characteristics of PMMA during accelerated aging.

## 2 Materials and methods

### 2.1 Sample preparation

**Fig 1:**
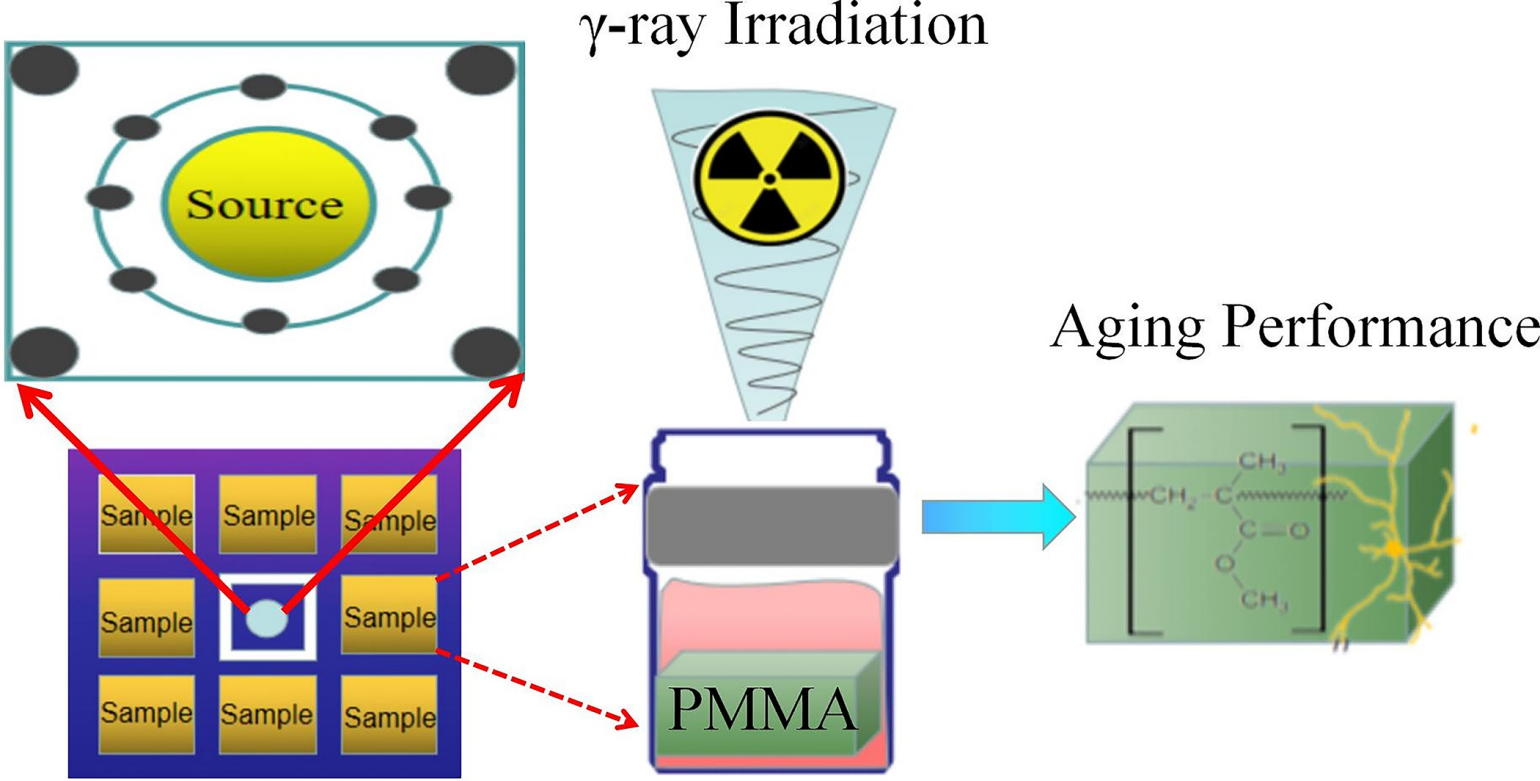
Schematic of the 60Co γ irradiation of the PMMA samples.

Commercial PMMA (model brand: Shengmei) was selected as the target material. All the specimens were 35 mm in length, 20 mm in width, and 5 mm in thickness. Before irradiation, the PMMA surface was cleaned with deionized (DI) water to remove impurities and ensure the reliability of the experimental measurement data. The PMMA samples were prepared at room temperature under different media conditions, including air and DI water immersion. A suitable clean and clear glass bottle that was sufficiently large to hold the PMMA sample was prepared. The sample should be completely immersed in the infiltrating liquid inside the bottle, and the bottle should be securely sealed to ensure that the liquid would not leak out (Fig 1).

### 2.2 Sample platform

The PMMA samples were irradiated with different doses (50, 250, 500, and 1000 kGy) of ^60^Co γ radiation at room temperature. The irradiation experiments were conducted at Sichuan Golden Radiation Co., Ltd. The dose rate was approximately 0.8 kGy/h. Irradiation was measured with a dosimeter during the irradiation process, and the error range of the doses was less than 5%. The samples were kept at room temperature during irradiation and then placed in a cool environment at 25°C after irradiation. The irradiated and unirradiated samples were characterized in the experiments.

1. **Surface morphology**
  The surface morphology of PMMA after exposure to different 60Co-γ doses was observed on a DM 1500 optical microscope. The grayscale values of the samples were also counted with ImageJ software.
2. **Light transmittance**
  The transmittance of PMMA exposed to various doses of 60Co-γ radiations is characterized by using UV 3600I plus 200-2500nm, according to GB/T2014-2008.
3. **Fourier transforms infrared absorption spectrum (FT-IR)**
  An infrared absorption spectrum of PMMA exposed to different media at different dosages was characterized by a NICOLET iS10 (Thermos scientific) infrared spectrometer.
4. **Yellow degree**
  The yellow degree is used to determine the amount of change in the properties of a material after irradiation. The degree of yellowness was determined with the NH300 yellowness meter according to ASTM-E 313–73 (D 1925).
5. **Contact angle meter**
  Characterization of hydrophobic changes of samples before and after irradiation using an optical contact angle measuring instrument (SDC-350), according to GB/T30693-2014.
6. **Shore stereoscope hardness**
  According to GB/T 1040–1992, the Shore stereoscope hardness of PMMA after exposure to 60Co-60Co-γ for different durations is tested by the TIME5430 testing machine.
7. **Characterization of glass transition temperature (Tg)**
  PMMA at different 60Co-60Co-γ doses was measured with a TA/DSC25 differential scanning calorimeter. According to GB/T 19466.2–2004, the DSC curve was performed at a temperature increment of 10˚C/min from 20˚C to 400˚C, and the intermediate temperature was regarded as the glass transition temperature.
8. **Gaseous phase gel permeation chromatography (GPC)**
  The relative molecular mass of PMMA after exposure to 60Co-60Co-γ for different dosages is characterized by PL-GPC50. Exposure to γ-ray irradiation and characterization of the relative molecular masses of PMMA under different dose irradiation intensities on the medium with THF.
9. **Scanning electron microscope**
  Samples were coated with gold and SEM images were obtained at an accelerated pressure of 15 kV. Afterward, the microstructure of the samples was characterized by scanning electron microscopy (SEM; tungsten scanning JEM [JSM-IT500]).

## 3. Characterization and testing

### 3.1. Surface morphology analysis of the samples

**Fig 2:**
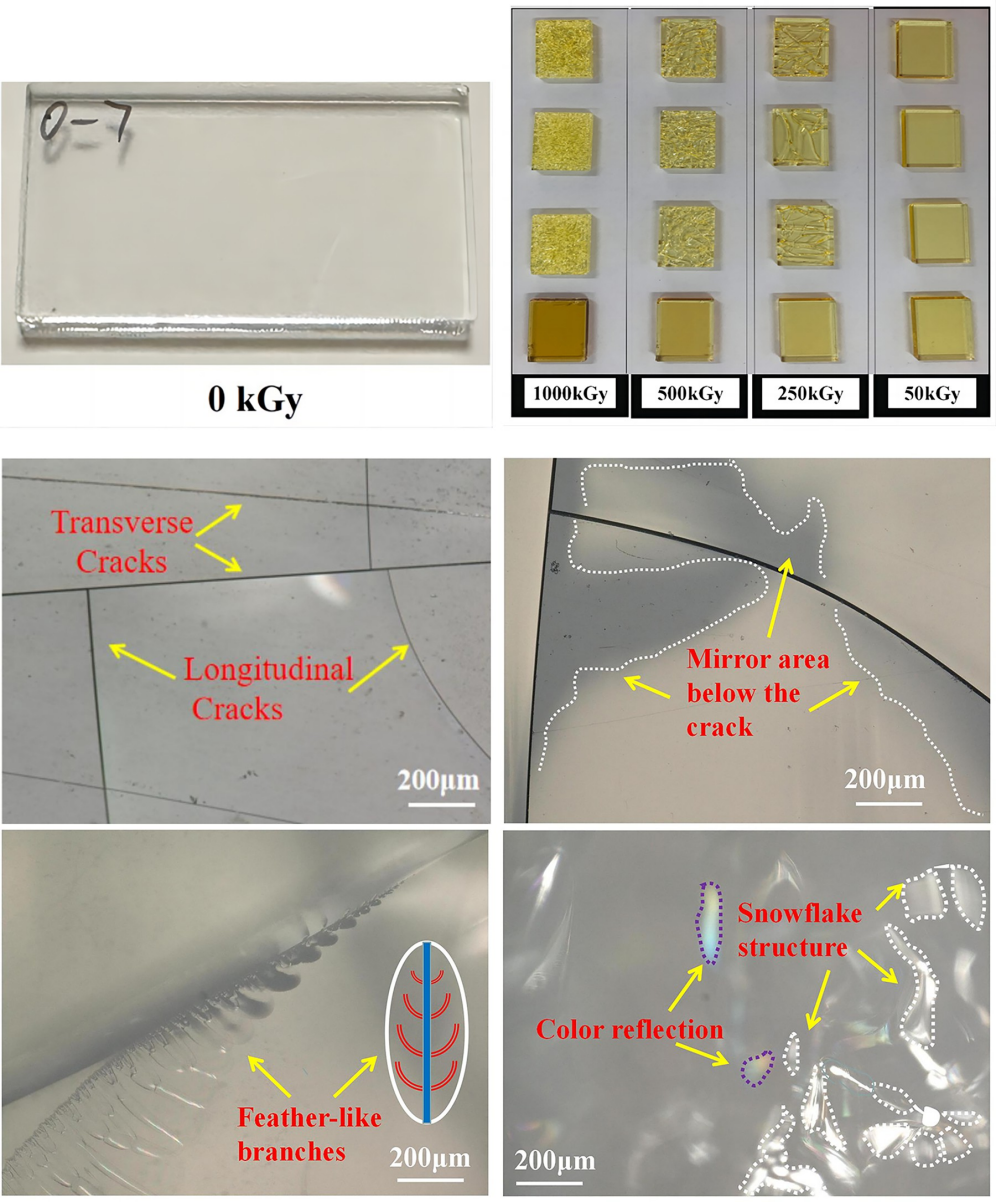
Morphology of PMMA specimens under different irradiation doses. 2.(a)-1 the whole structure before irradiation, 2(a)-2 the whole structure after irradiation, (b)-(e): typical crack changes under light microscope.

The changes in the color of the PMMA material after γ-ray irradiation can directly respond to the aging of the material. When the radiation dose increases, the material color differences become larger. The Fig 2(A) shows the appearance of the material after irradiation. Compared with the pre-irradiation, the integrity of the material is good, and with the change of irradiation dose, the material undergoes changes in color and cracks.The surface morphological features of the original samples and the ^60^Co γ-irradiated samples at various doses (50, 250, 500, and 1000) kGy were investigated via optical microscopy. Typical cracks on the irradiated samples are shown in Fig 2B–2E. The aging failure of the material generally increases with an increase in irradiation dose [21]. From this experiment, the PMMA material starts to show surface cracks when aging occurs, and the cracks exhibit uneven thickness and irregular direction. Crack expansion seems to start under the main crack of the material, below which the PMMA material appears as a mirror area. The area extends and branches out like a feather. The crack increases as the surface roughness of the material increases. As irradiation dose continues to increase, a snowflake-like structure appears, with irregular separation between cracks. Cracking is assumed to be cause by internal stress [22, 23].

**Fig 3:**
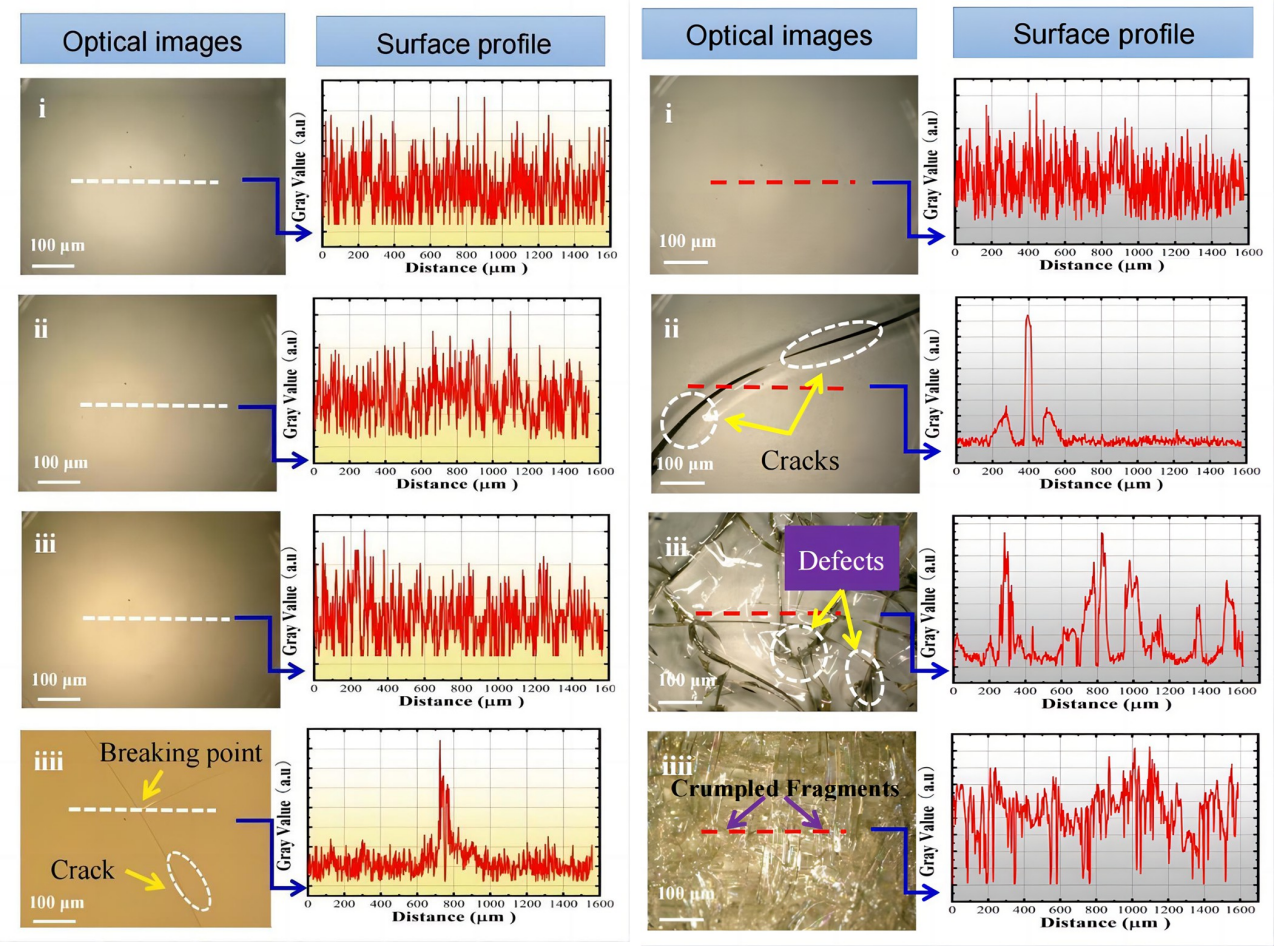
The surface images of PMMA irradiated by 60CO γ. (a) in air medium, (b) in DI water medium. optical images: PMMA under irradiation with increasing γ-ray dose from top to bottom, surface profile: the selected white dashed line in optical images (modified the pictures).

The images taken with an optical microscope were analyzed with the open-source ImageJ software [24]. Fig 3 shows the surface images of the sample irradiated by ^60^CO γ. As depicted in Fig 3(A)(i), the sample is smooth and homogeneous, without visible cracks. In Fig 3(A)(i–iiii), the color of the surface of the samples irradiated by immersion in air ranges from light to dark, indicating the aging effect on the color of the samples due to an increase in irradiation dose. When the irradiation dose reached 1000 kGy [Fig 3(A)(iiii)], cracks were observed on the sample surface in different directions, along with crack intersections. From the subsequent changes in the sample, such intersections may be inferred as the rupture points where pinholes, defects, or ruptures occur. ^60^CO γ radiation affected the sample by increasing the dose, and cracks varied and combined with other morphological changes. Observation of the samples infiltrated with DI water indicated that the irradiation dose of 250 kGy produced significantly broader and deeper cracks than the air-irradiated samples, as reflected in the visible appearance of the cracks, which became thicker with a darker color [Fig 3(B)(ii)]. When irradiation dose continued to increase to 500 kGy [Fig 3(B)(iii)], the cracks widened and pinholes appeared. When pinholes and cracks fuse, a sample is fractured. When irradiation dose reached 1000 kGy [Fig 3(B)(iiii)], sample integrity was still maintained. However, the crumpling phenomenon occurred inside the sample, reflecting a colorful appearance. Internal changes, such as small fragments (one next to another, similar to crumpled paper), could be observed. These changes may be attributed to the increase in the number of small cracks under the main crack after accelerated aging and the formation of new structures between cracks. These phenomena delayed the expansion of the main crack and changed internal stress conduction to a certain extent, and thus, the sample temporarily did not crumble.

By collecting and counting the surface morphology of the samples, we studied the surface changes of the irradiated samples by using the open-source ImageJ software. We determined that the samples’ surface profile (white dashed part) exhibited corresponding changes with increasing dose. The gray scale of the samples irradiated in air medium did not present significant changes. Only when irradiation dose reached 1000 kGy did the gray scale increased significantly at the location where cracks were created, and the contours were changed as shown in Fig 3(A). The gray scale of the samples irradiated in DI water medium increased with irradiation dose. As depicted in Fig 3(B), the increase in gray scale further confirmed the gradual deepening of cracks, fracture defects, and pinholes. The grayscale analysis (from top to bottom) of the samples exposed to higher doses also indicated that aging changes on the surface occurred due to high irradiation doses.

### 3.2. Transparence analysis

**Fig 4:**
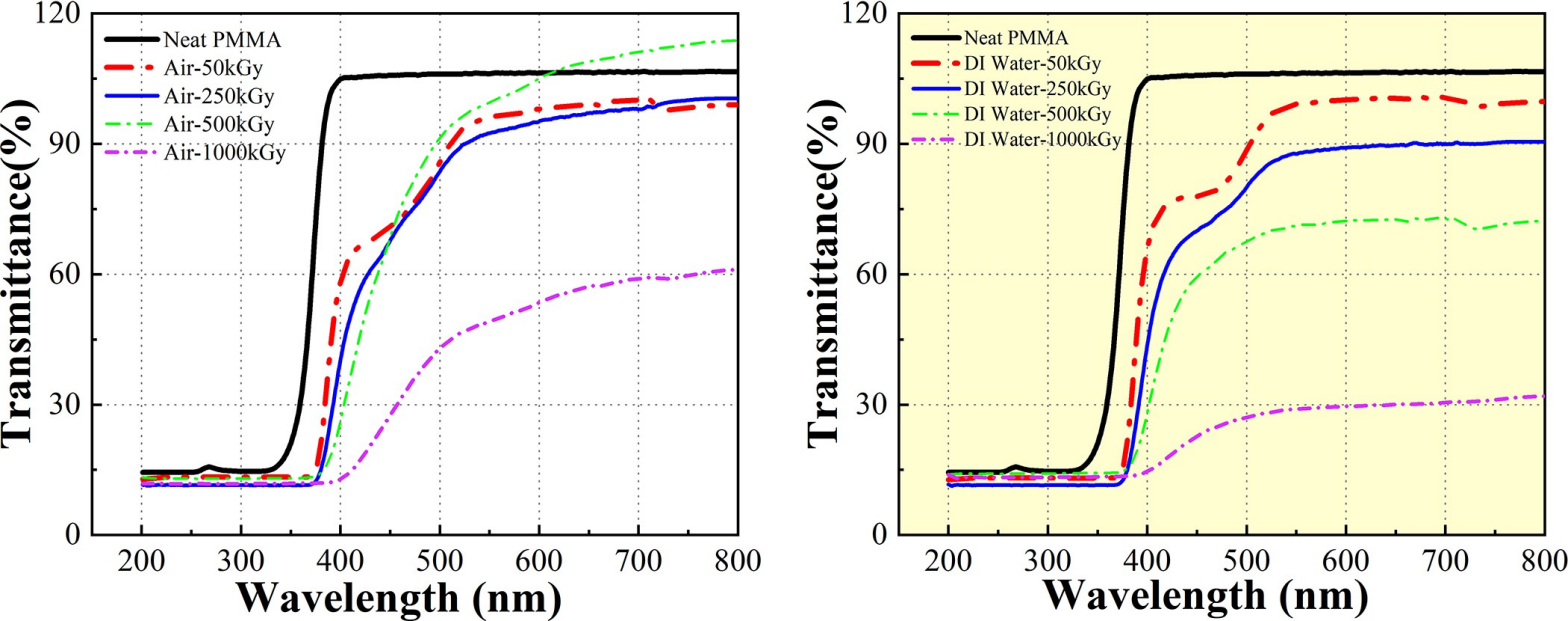
Evolution of transmittance with various 60Co γ irradiation doses for PMMA. (a) in air medium, (b) in DI water medium.

**Fig 5:**
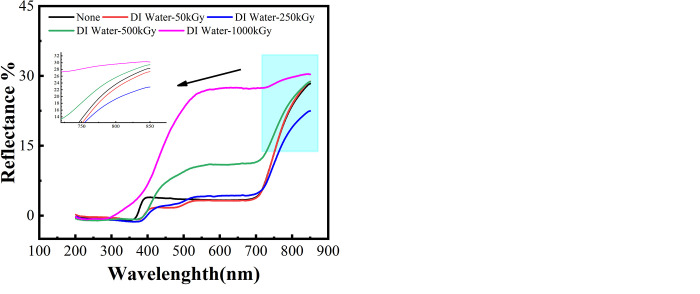
Analysis of PMMA reflectance at different 60Co γ irradiation doses. (a) in air medium, (b) in DI water medium.

PMMA is currently the best transparent polymer material, with a light transmission rate of 92%. As an ophthalmic biomaterial, the change in its light transmission rate is an essential indicator for determining the good performance and sustainability of materials [25]. Observing the change in the light transmission rate of PMMA in different media before and after aging treatment under extreme conditions can provide valuable information. The light transmission rate of PMMA after irradiation is shown in Figs 4 and 5. Transparency does not change significantly, indicating that the effect of ^60^CO γ irradiation on PMMA is limited. During pre- and post-irradiation treatment in air medium, a decrease in the transmittance of PMMA is observed at different irradiation doses, except for the increase in transmittance at an irradiation dose of 500 kGy. Nevertheless, transmittance is still higher than 80% at irradiation doses of 250 kGy and 50 kGy. When irradiation dose reaches 1000 kGy, transmittance decreases significantly to 50%. In DI water medium, the light transmittance of all the irradiated samples decreases, and light transmittance is only about 20% at the maximum ^60^CO γ irradiation dose.

We can conclude that the decrease in visible light transmission of PMMA irradiated in DI water medium is more significant than that in air medium. In particular, 400–500 nm light transmission increases rapidly and reaches saturation. Such results indicate that ^60^Co γ radiation exerts the most significant effect on the light transmission of PMMA material and continues to reach saturation within the visible range. Within the UV range, however, scattering visible light and absorbing UV light play a dual role due to the dual effect of irradiation on the material, causing a decrease in visible light transmission and an increase in UV light absorption of the material after irradiation.

### 3.3. IR absorption spectrum and related analysis of PMMA

**Fig 6:**
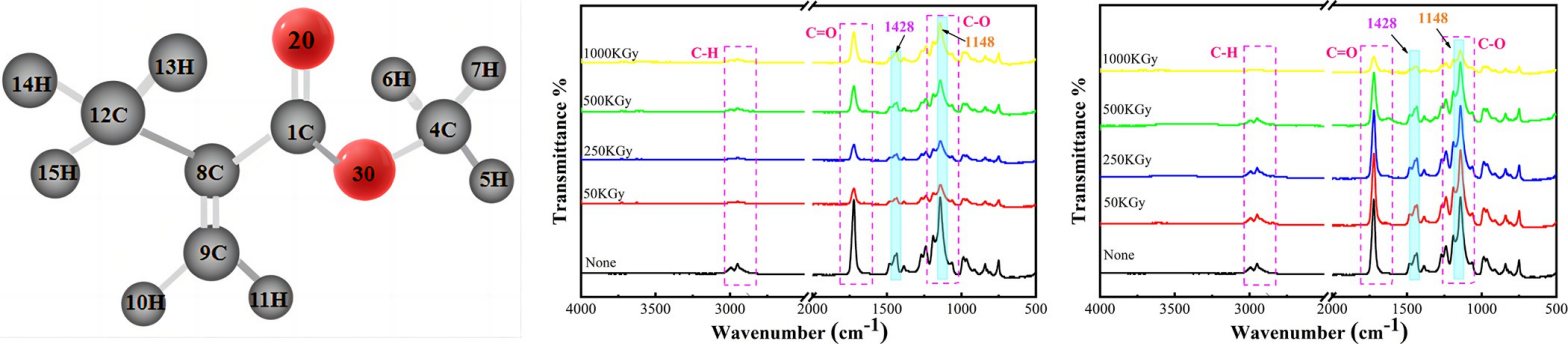
IR spectral analysis of PMMA exposed to different doses of 60Co γ irradiation. (a) the monomer molecular structure of PMMA, (b) the IR absorption spectra of PMMA in air medium before and after 60Co γ irradiation, (c) the IR absorption spectra of PMMA in DI water medium before and after 60Co γ irradiation.

PMMA is wildly used in many ocular implants and contacts, which is a classic polymer biomaterial has stable structure-as shown in Fig 6A. As a biomaterial, the physicochemical properties of PMMA have been well proved, and the interfering factors can be excluded in this study, which is beneficial to our research.When polymer materials irradiated, their macromolecular chains and chemical bonds may change, thus affecting their mechanical, physical properties, therefore infrared spectroscopy tests were carried out in our study. Fig 6B and 6C show the IR absorption spectra of PMMA in different media before and after ^60^Co γ irradiation. As depicted in the figures, the degradation reaction of PMMA includes the degradation of ester and methyl side groups and the random degradation of the main chain. The groups in the degradation products include O-H (3441 cm^−1^), C-H (2977 cm^−1^, 2953 cm^−1^), C = O (1730 cm^−1^), and C-O (1148 cm^−1),^ which are the same as the absorption peaks in the PMMA molecular chain before degradation [26]. No new absorption peaks are observed in the IR absorption spectrum of PMMA after degradation; however, a change in the intensity of the characteristic peaks occurs. The positions of the characteristic absorption peaks in the IR absorption spectra do not change, and no new characteristic absorption peaks appear. This result indicates that no new groups are generated in the main chain after irradiation [27].

**Fig 7:**
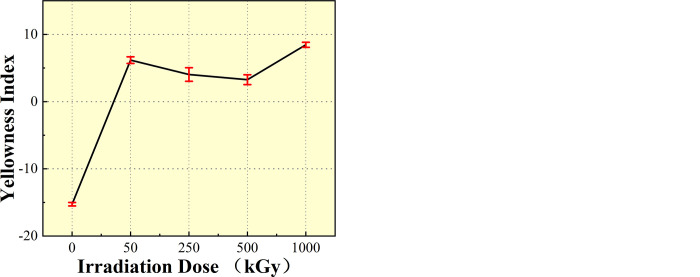
Dependence of the yellowness index on various doses of 60Co γ irradiation. (a) in air medium, (b) in DI water medium.

The characteristic absorption peaks of ^60^Co γ are visible pre- and post-irradiation in different media [28], where the high-frequency band characteristic peaks 1148 cm^−1^ and 1428 cm^−1^ are visible (shown in blue boxes), and no coverage of the ^60^Co γ characteristic peak occurs as suggested in the literature [29]. The irradiation dose and interaction of different media exert a common effect on PMMA and do not cause strong PMMA absorption. Fig 6B shows the IR absorption spectra of PMMA before and after irradiation in an air medium. The peak around 1740 cm^−1^ is the peak of the C = O bond, which decreases at 50 kGy and 250 kGy irradiation doses, but increases after irradiation dose continues to increase. The C-O bond cleavage increases at moderate dose aging and recombines at high dose irradiation aging. The peaks at 1300–1100 cm^−1^ are the expansion and contraction of the C-O bond. The vibration peak also exhibits the exact change as the C = O bond, proving that the change in C and O structures is not simply a linear change influenced by irradiation dose, but a reaction in which O in the air is involved in the continuous change of cleavage and polymerization. Fig 6C shows the IR absorption spectrum of PMMA before and after irradiation in DI water medium. The primary difference between Figs 6B and 7C is that the intensity of the characteristic absorption peak decreases linearly with increasing irradiation dose. The ^60^Co γ intensity of the characteristic peak decreases only at an irradiation dose of 1000 kGy, while the other irradiation doses exert no significant effect on peak intensity.

This finding indicates that ^60^Co γ irradiation itself does not significantly affect the PMMA structure. Moreover, the double effect of irradiation and the medium does not cause the creation of new groups or the complete disappearance of the original groups. However, by comparing the changes in the two figures, the components in the air medium involved in the irradiation effect on PMMA are not simply cleavage reactions. In the case of DI water medium, DI water produces cleavage in an irradiation environment, and thus, cleavage intensity is more significant with an increase in irradiation dose. After PMMA aging, the breakage of methyl and ester groups produces small molecules, such as CO, CO_2_, and CH_4_; these chromophores cause the yellow color of the aged samples [30].

### 3.4. Yellowness index

After aging with ^60^Co γ, the PMMA materials were subjected to colorimetric testing by using a yellowness meter. Aging in a thermal environment will make the polymer material black and yellow. Three different locations of color measurement points were selected for each aged specimen, and the average value was taken. As aging progresses, the yellowness index increases while the whiteness index decreases. As shown in Fig 7, the yellowness index of the irradiated samples exhibited a gradual increase in air medium. By contrast, the yellowness index decreased slightly in DI water medium at irradiation doses of 250 kGy and 500 kGy. However, it demonstrated a higher rise as irradiation dose increased. At smaller irradiation doses, the yellowness of PMMA was inevident in both media. When dose increases to a certain extent, air and DI water start to interact with the molecular structure of PMMA and appear to transform part of the radiation energy into heat emitted out, such that yellowness does not change considerably at intermediate irradiation doses [31]. An analysis can be obtained from the irradiation dose of the most significant PMMA samples, whether in air or DI water medium. The aging degree is the highest, and the process is also the fastest, indicating that its aging rate is more significant than those of the other irradiation doses.

**Fig 8:**
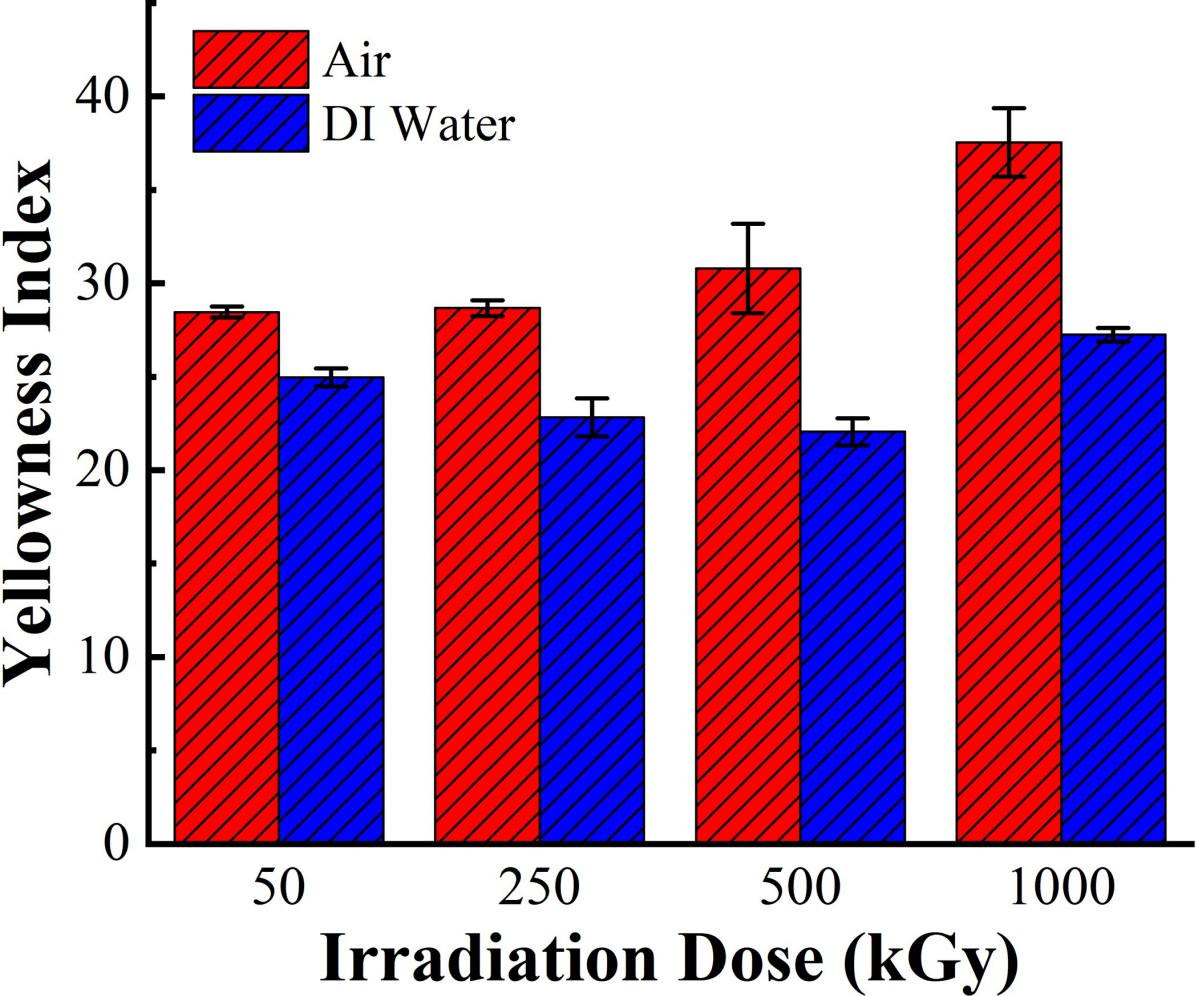
Comparison of yellowness changes in PMMA after 60Co γ irradiation in the two media.

Fig 8 shows that the overall yellowing of irradiated PMMA in air medium is higher than that in DI water medium, indicating that the addition of DI water slows down the aging of the PMMA material under the same irradiation conditions and plays a role in inhibiting the yellowing of the material.

### 3.5 Contact angle analysis

**Fig 9:**
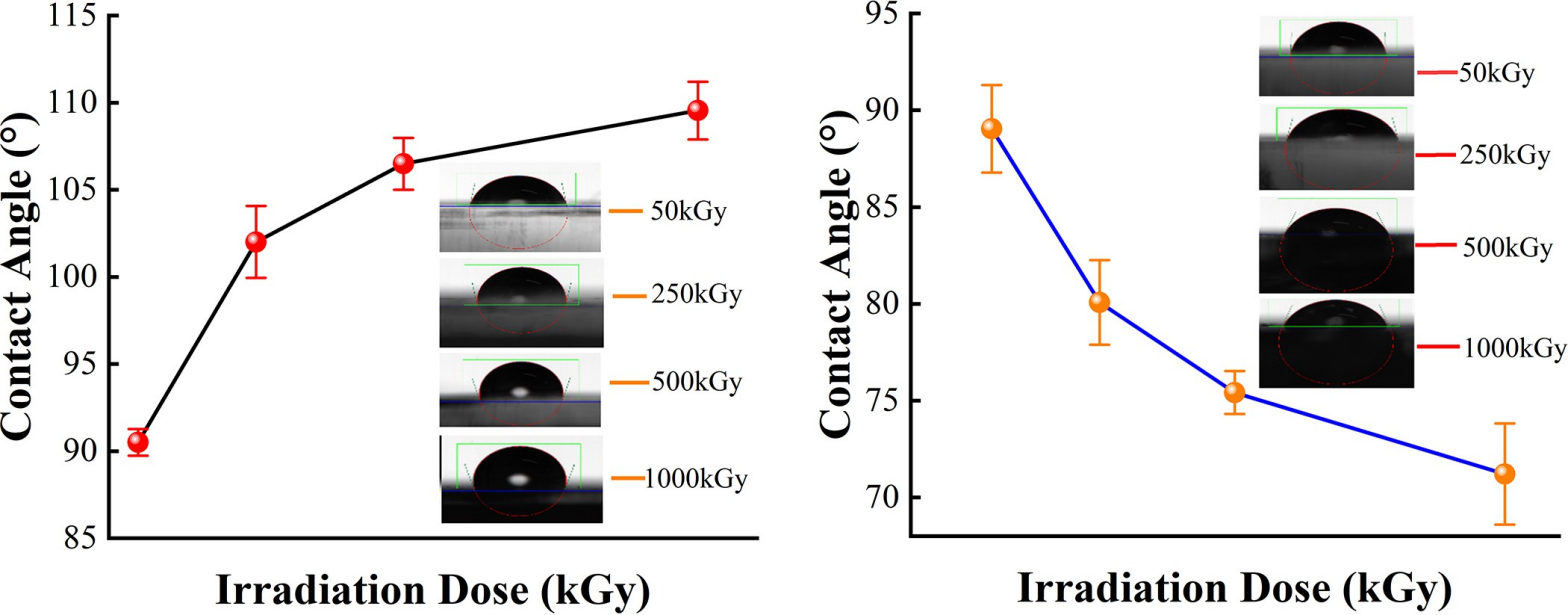
Tendency of contact angle changes of PMMA after 60Co γ irradiation. (a) in air medium, (b) in DI water medium.

**Fig 10:**
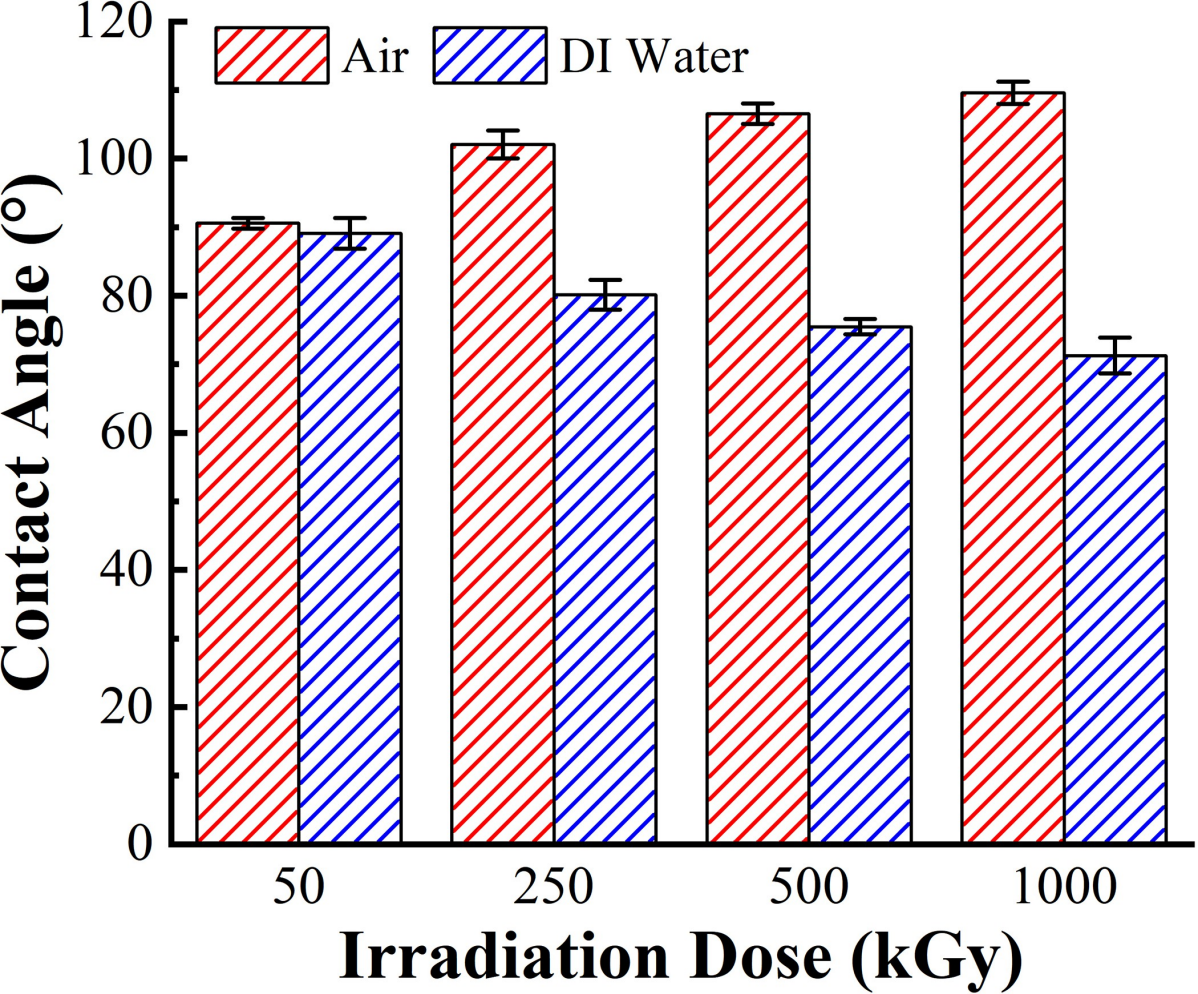
Comparison of the contact angle in PMMA after 60Co γ irradiation in two media.

Fig 9 shows the observation of the surface wettability of the irradiated PMMA samples in different media and their adhesion properties. From the Wenzel equation, when the contact angle is less than 90°, surface roughness increases to make the contact angle smaller and wettability better [32]. Through the experiments, we determined that the contact angle of the irradiated samples in both media was less than 120°, proving that irradiation did not change the hydrophobicity of the PMMA material. The contact angle of the PMMA material in both media does not change significantly when ^60^Co γ irradiation dose was 50 kGy. However, the contact angle of the PMMA material in air medium gradually increases with an increase in irradiation dose. In DI water medium, contact angle gradually decreases. Simultaneously, the difference in contact angle of the PMMA materials in both media increases with an increase in irradiation dose (in Fig 10), demonstrating that ^60^Co γ affects the change in wettability of the material. The change in medium modifies the material’s surface. In air medium, the contact angle of the material becomes larger and develops in the hydrophobic direction, wettability becomes worse, and adhesion weakens. In DI water medium, the contact angle of the material becomes smaller and develops in the hydrophilic direction, wettability is enhanced, and adhesion is strengthened.

### 3.6. Hardness analysis

**Fig 11:**
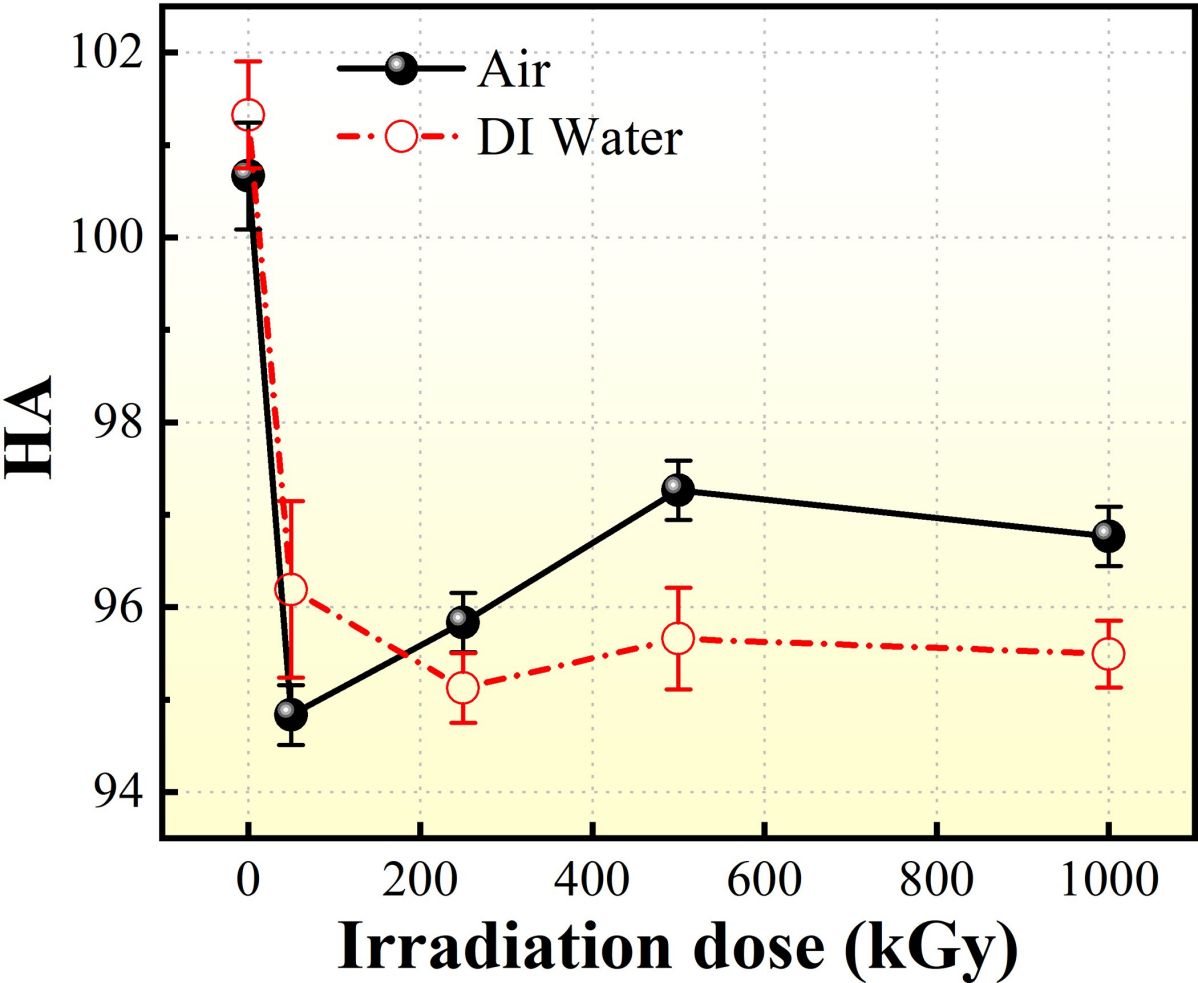
Shore hardness value of 60Co γ-irradiated PMMA impregnated with two media.

Fig 11 shows the effect of different ^60^Co γ irradiation doses on the hardness of PMMA materials in the two media. In previous studies, Peuget used different types of ion-irradiated glasses to simulate changes in glass properties under irradiation conditions and noted that the hardness of the cured glass decreased after ion irradiation [8, 9]. Wang et al. [13, 14] used nano-hardness tests to obtain the effects of irradiated borosilicate glass hardness decrease after irradiation, among other effects [33]. The mechanical strength of PMMA is high. No study on the effect of high irradiation dose on PMMA material has yet been conducted. In the current experiment, as dose increased from 50 kGy to 1000 kGy, the PMMA material was subjected to accelerated aging treatment. The Shore hardness (HA) of the material did not change significantly under irradiation in different media. The surface hardness of the irradiated samples decreased by <5%. However, the hardness of the material decreased when compared with the pre-irradiated PMMA samples. When irradiation dose was 50 kGy, the effect in air medium was greater than that in DI water medium. As irradiation dose increased to 500 kGy, the hardness of the material was basically saturated in air medium. Meanwhile, in DI water, hardness appeared to increase after a short, abrupt drop in irradiation dose, while hardness decreased at the maximum irradiation dose. The data show that the hardness of the PMMA material after ^60^Co γ irradiation is generally greater in air medium than in DI water under extreme aging conditions (irradiation dose of 1000 kGy). By contrast, the aggregate decrease in surface hardness of the samples made from ^60^Co γ irradiated material was significant.

### 3.7. Comprehensive temperature analysis

**Fig 12:**
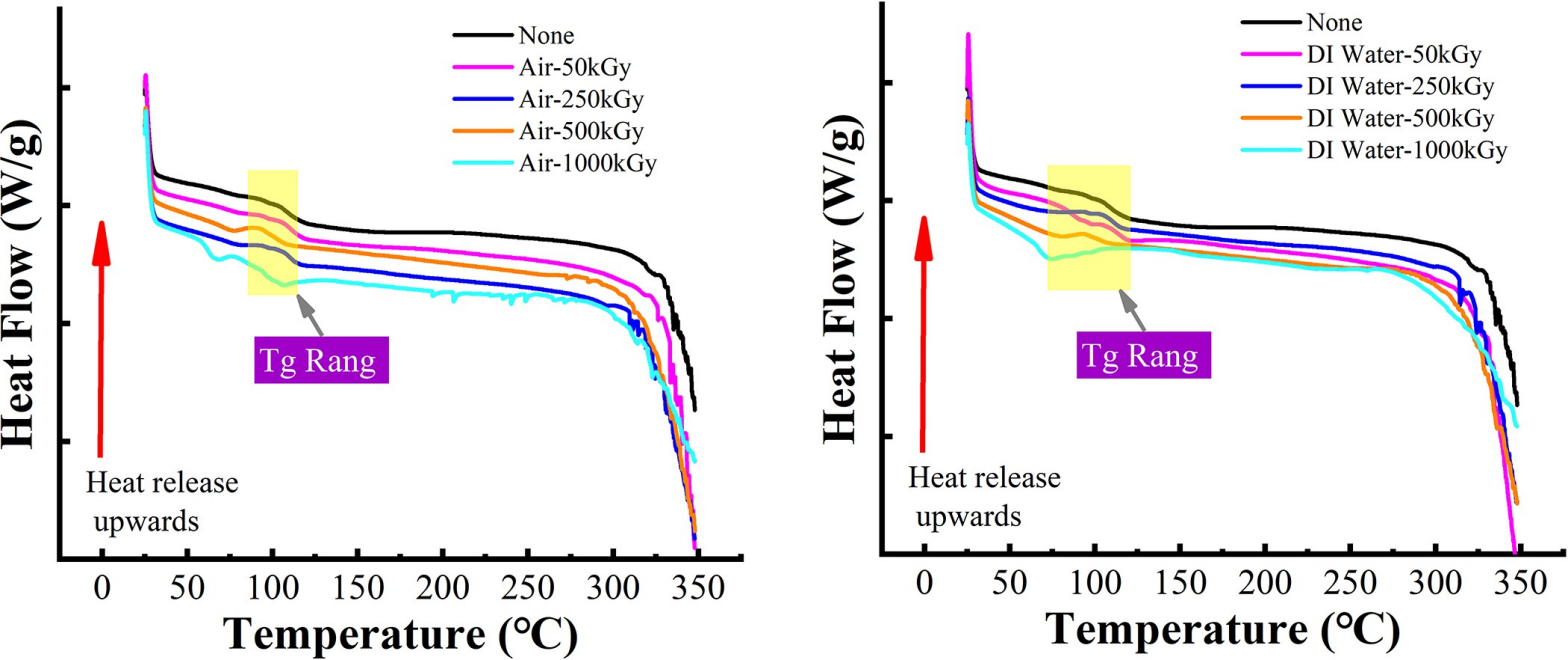
DSC curves of PMMA under different doses of 60Co γ irradiation. (a) in air medium, (b) in DI water medium.

**Fig 13:**
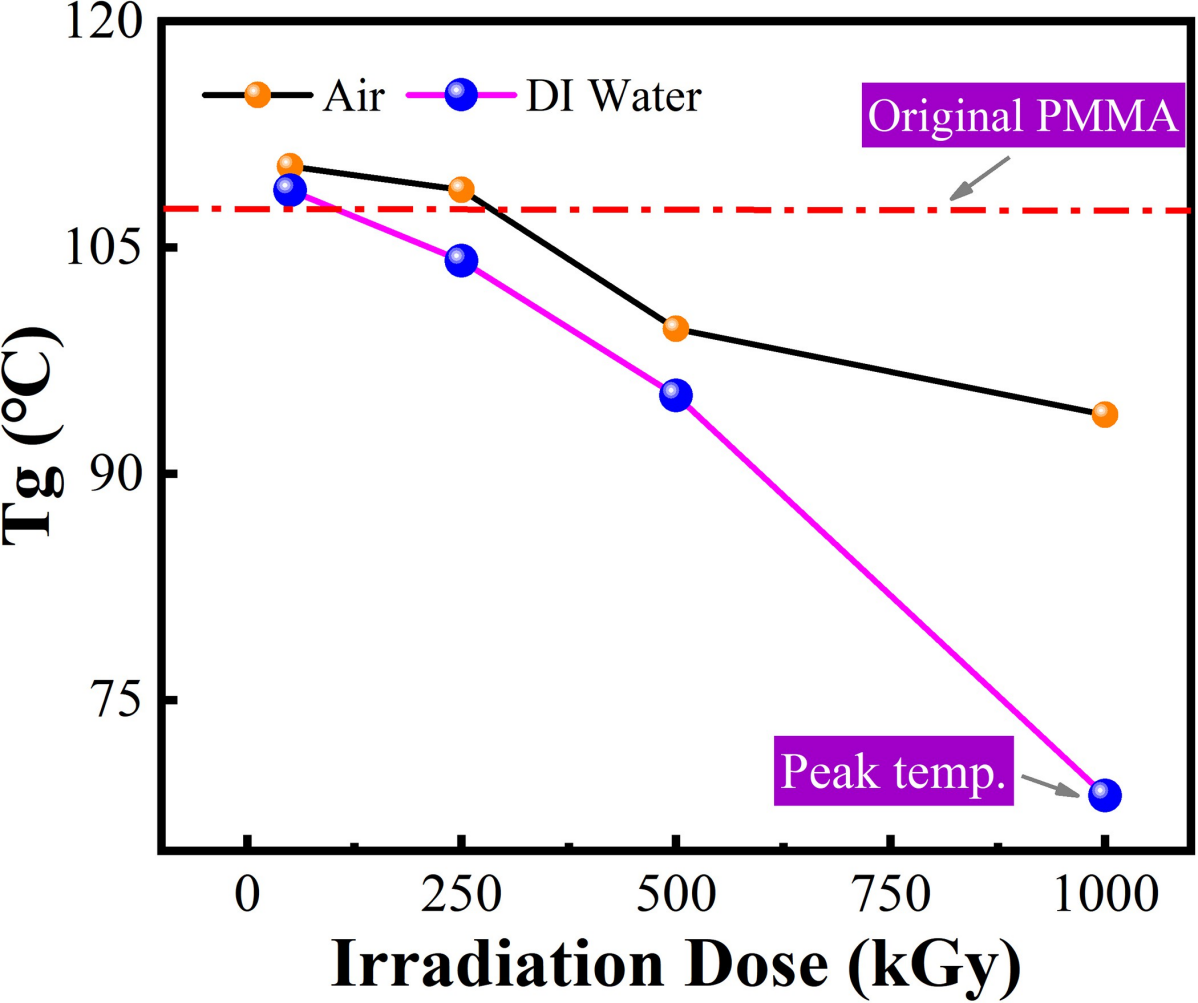
Variation of glass transition temperature in PMMA after irradiation.

Fig 12 shows the DSC results of PMMA after irradiation in two media at different ^60^Co γ irradiation doses. In accordance with GB/T 19466.2–2004, the glass transition temperature of PMMA can be determined as listed in Table 1 and Fig 13. As shown in Fig 12, the DSC curves of PMMA after ^60^Co γ irradiation exhibited the same trend and direction. The addition of air and DI water during irradiation did not significantly change the thermodynamic properties of the PMMA material. From the yellow boxed part combined with the data in the table, we can obtain Fig 13, which shows the glass transition temperature of PMMA after irradiation in two media at different times. Although the DSC paths are similar in Fig 12, a magnified analysis shows that the glass transition temperature of the irradiated PMMA material is decreasing compared with the unirradiated PMMA (the red dotted line shows the glass transition temperature of PMMA in Fig 13, which is approximately 105°C). In DI water medium, the glass transition temperature of irradiated PMMA decreased more with increasing irradiation dose, and peak temperature appeared when irradiation dose reached 1000 kGy. In general, a change in the relative molecular mass of a polymer leads to a change in its glass transition temperature [34].

**Table 1:** Thermal analysis data of PMMA material after 60Co-γ irradiation.

Polymer Type	Irradiation time (kGy)	Tg (°C)	Δcp (J/(g.°C))
**Neat PMMA**	0	108.43	0.335
	50	110.45	0.311
**Air**	250	108.89	0.304
	500	99.68	0.267
	1000	93.98	0.395
	50	114.18	0.202
**DI-Water**	250	109.51	0.265
	500	100.60	0.154
	1000	74.07	/

### 3.8. Relative molecular mass on the surface of PMMA

**Fig 14:**
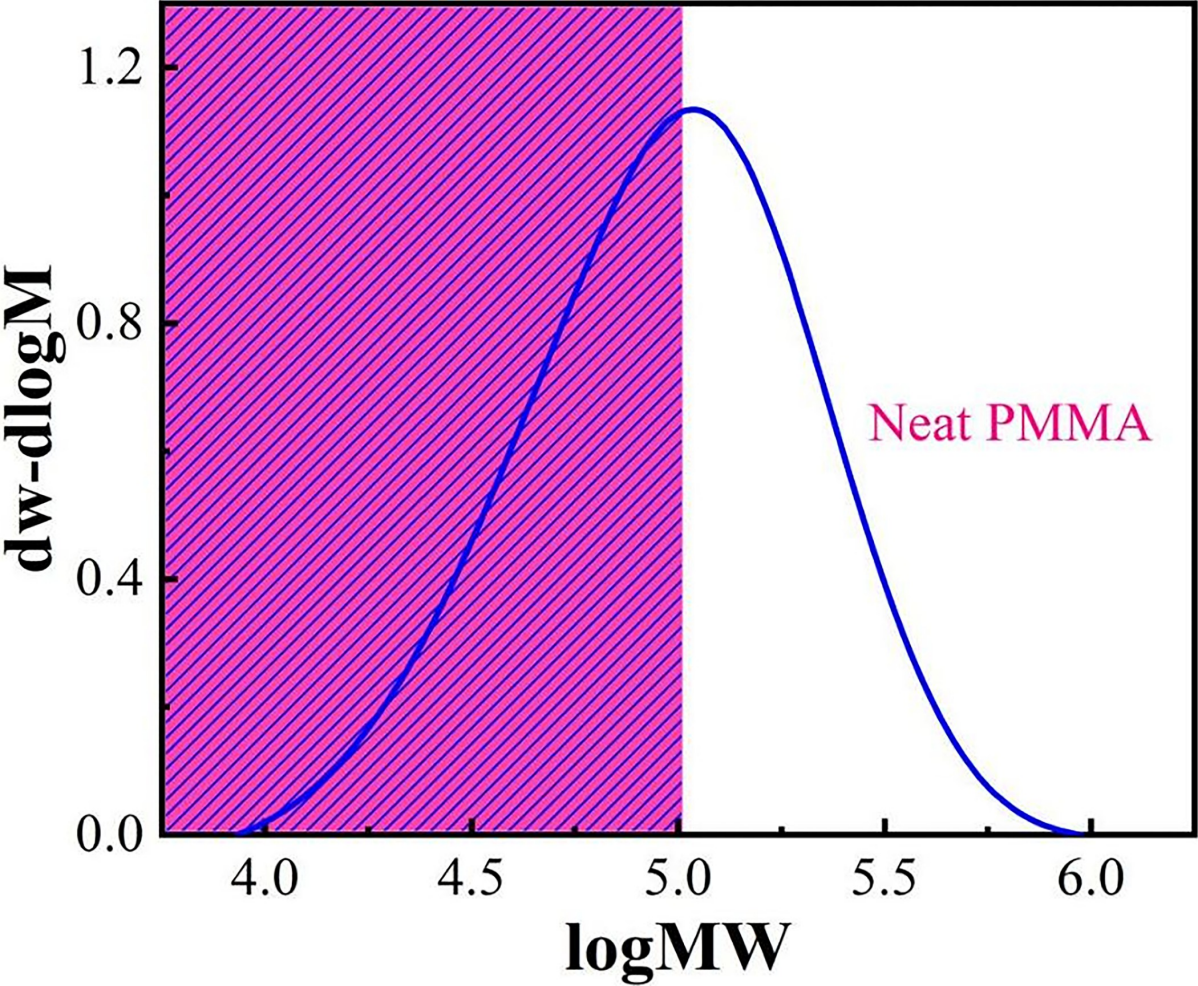
Relative molecular mass differential distribution curves of PMMA.

**Fig 15:**
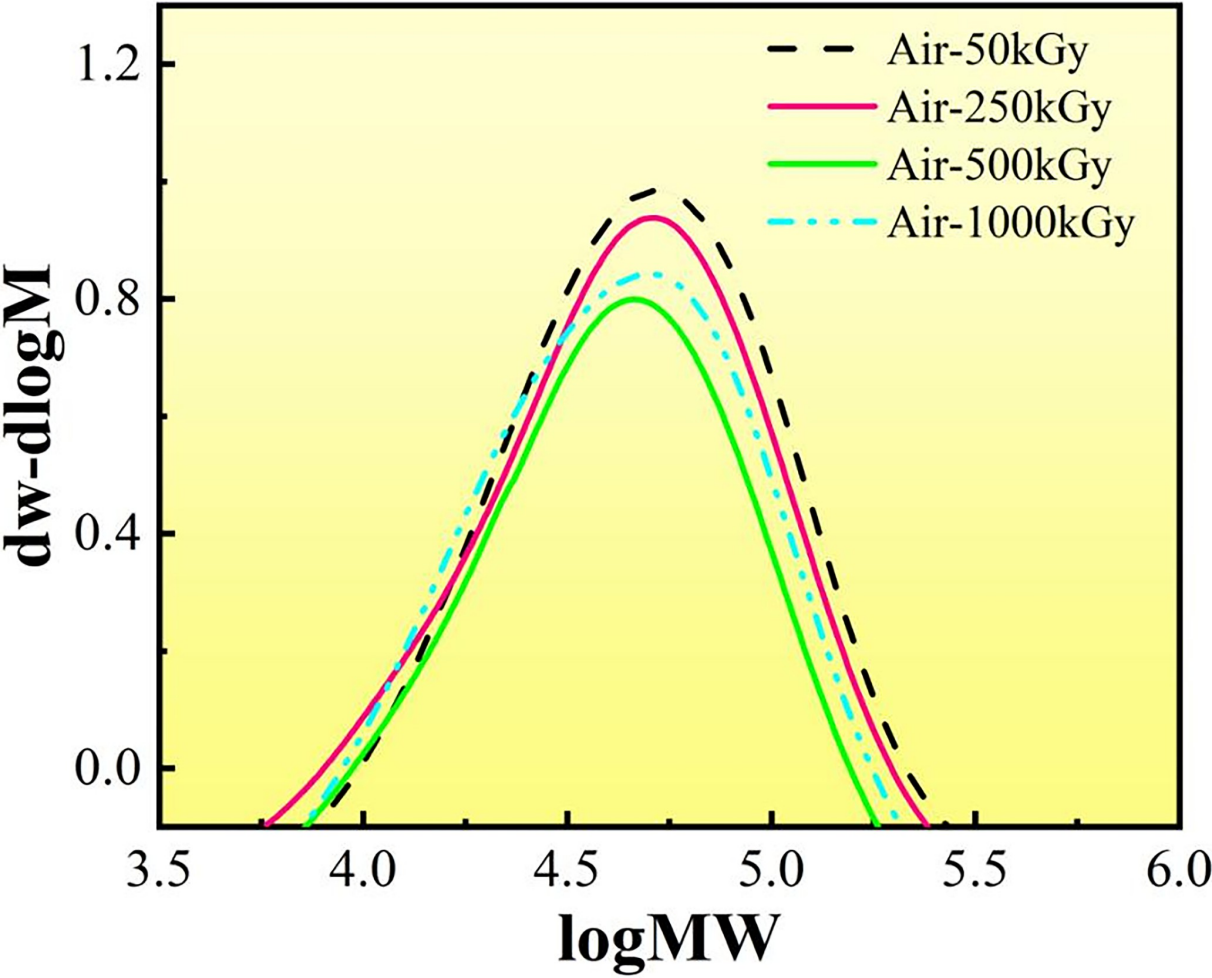
Relative molecular mass differential distribution curves of PMMA under air.

The influence of molecular weight distribution on the performance of polymer materials is primarily expressed in the range of high and low molecular weight parts and their contents. The average molecular weight of a polymer material is the basis for determining the application range of the material. Fig 14 shows the mass fraction of unirradiated PMMA, and the mass fraction is almost sinusoidally distributed. The GPC data of PMMA in both media at different irradiation doses are referred to Tables 2 and 3. In DI water media, the MP value decreases with increasing irradiation dose, indicating a significant change in the molecular mass, which is the most important percentage of the whole material, showing a significant decrease in molecular mass and demonstrating a gradual decrease in the relative molecular mass of PMMA, signifying that irradiation causes a break in the main chain of PMMA [35]. Mn is more reflective of the small molecules in the material because the smaller molecular weight leads to more molecular data, while Mw is more reflective of the large molecules in the material because the larger the molecular weight the heavier the individual molecules will be. From Tables 2 and 3, we see that irradiation reduces both large and small molecules in PMMA, regardless of the medium. As the irradiation dose increases, Mn decreases from 40012 to 5719 in air medium. the decrease is 85%, while in DI water, Mn decreases from 40475 to 5597. the decrease is 86%, a continuous decrease indicates that the degradation of PMMA continues to increase with the irradiation dose. From the values of PD coefficients, we found that the molecular weight distribution was dispersed in both media, indicating the presence of molecules of various molecular weights, presenting the small and large molecular activities [36]. (Fig 15)

**Table 2:** GPC data of PMMA after irradiation in air media.

Irradiation dose	Mp	Mn	Mw	PD value
**50kGy**	60846	40012	69361	1.733
**250kGy**	19274	10714	21744	2.029
**500kGy**	16502	9326	18043	1.93
**1000kGy**	9737	5719	10387	1.816

**Table 3:** GPC data of PMMA after irradiation in DI water media.

Irradiation dose	Mp	Mn	Mw	PD value
**50kGy**	66675	40479	78137	1.93031
**250kGy**	29469	21944	34605	1.57697
**500kGy**	18437	10602	20476	1.93133
**1000kGy**	9321	5597	9977	1.78256

### 3.9 SEM of cracks and fracture surfaces

**Fig 16:**
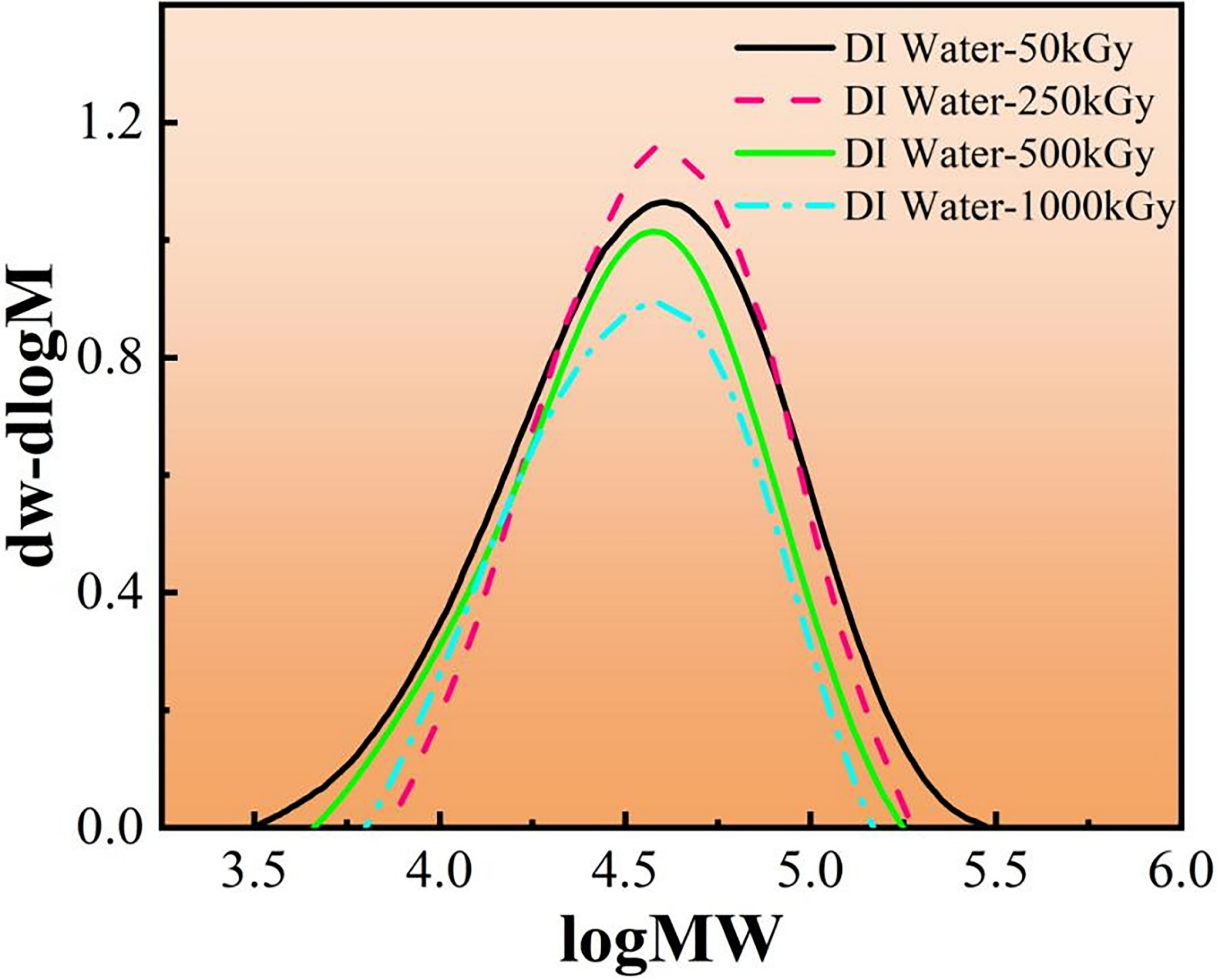
Relative molecular mass differential distribution curves of PMMA under DI water.

**Fig 17:**
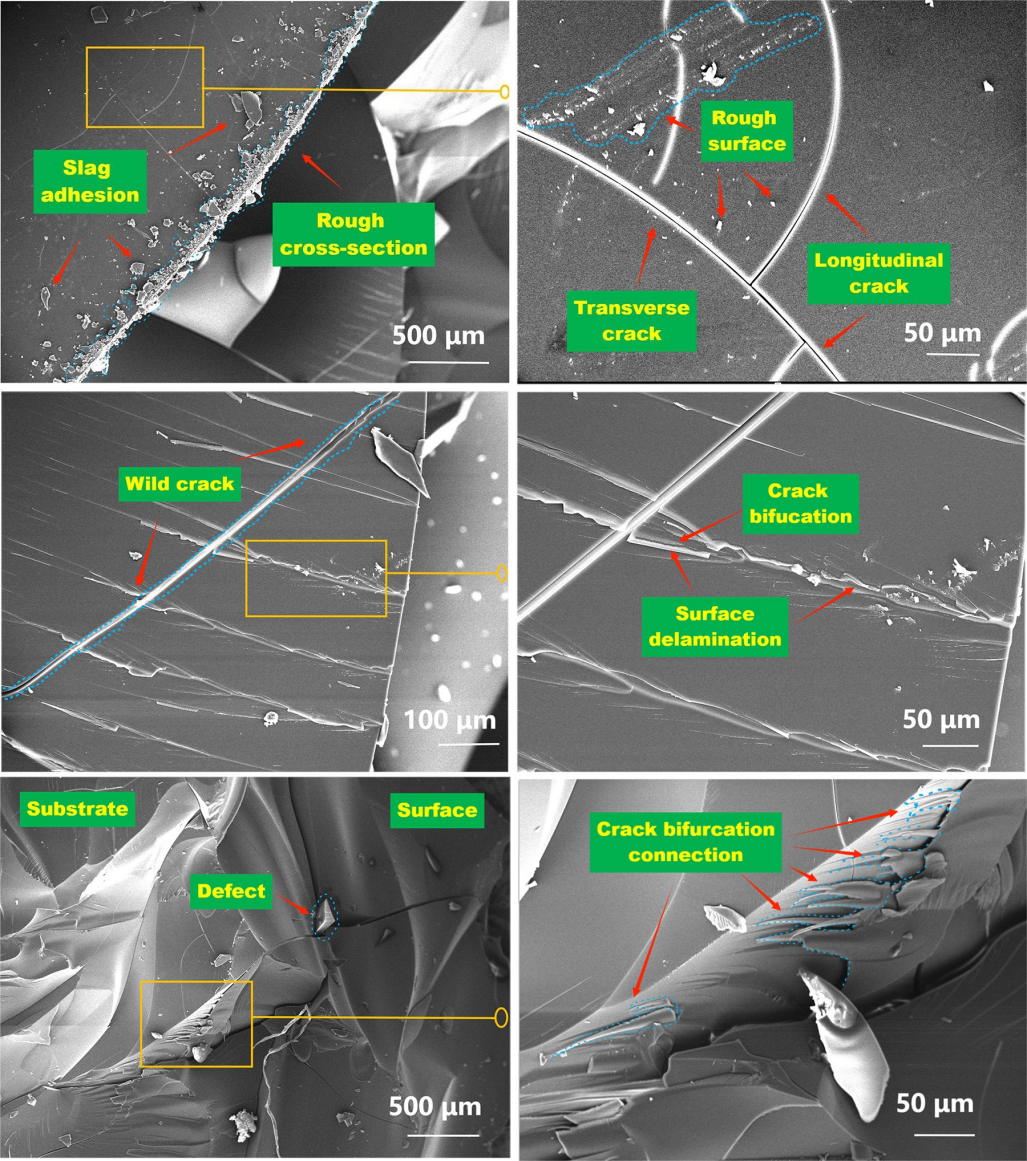
Scanning electron micrographs of PMMA after 60Coγ irradiation. (a)-(f): microscopic changes of different cracks under electron microscope (modified the pictures).

As irradiation and aging time increases, the internal structure of PMMA changes, causing PMMA to crack, become brittle, and even drop slag. When the medium is DI water, more fracture zones are formed on the surface of the material and cracks become denser. As irradiation dose continues to increase, the surface of the material becomes rougher and cracks expand and cross, causing further defects and damages. PMMA materials with different ^60^Co γ irradiation doses were observed under a scanning electron microscope. The surface roughness of the material increased significantly, while severe roughness of the fracture surface was observed, accompanied by adhesion of dropped slag. Transverse and longitudinal cracks appeared, exhibiting a relatively irregular state [Fig 17(A)]. A few cracks were not connected to the original crack and had stopped expanding, while others came in contact and crossed the main crack, forming a new crack zone and extending further to other locations. When two horizontal cracks intersect, the PMMA interface forms an isolated zone, which may lead to the formation and propagation of microscopic interfacial cracks at the interface, along with the formation of pinholes or fractures at the rupture point. As shown in Fig 16(C) and 16(D), branching and bifurcation occur as crack expands along the interface direction, causing the delamination of the interface surface and further increase in roughness. On the fracture surface of the failed material, crack extension bifurcation can be seen crawling over the interlayer material. Simultaneously, crack paths exist beneath the failed tissue. When the material fails under irradiation [Fig 17(C)], crack bifurcation can be seen crawling over the fracture surface [Fig 17(D)], near the end of the cracks. The surface and underlying structure of the material is clearly visible, with the surface layer appearing broken and the underlying layer visible as a haphazard crystal structure, with greater separation between layers due to the crack bifurcation that partially connects them, while new cracks can be observed below the fracture surface. Failure can be inferred to continue occurring at different levels as cracks appear.

## 4. Mechanism

**Fig 18:**
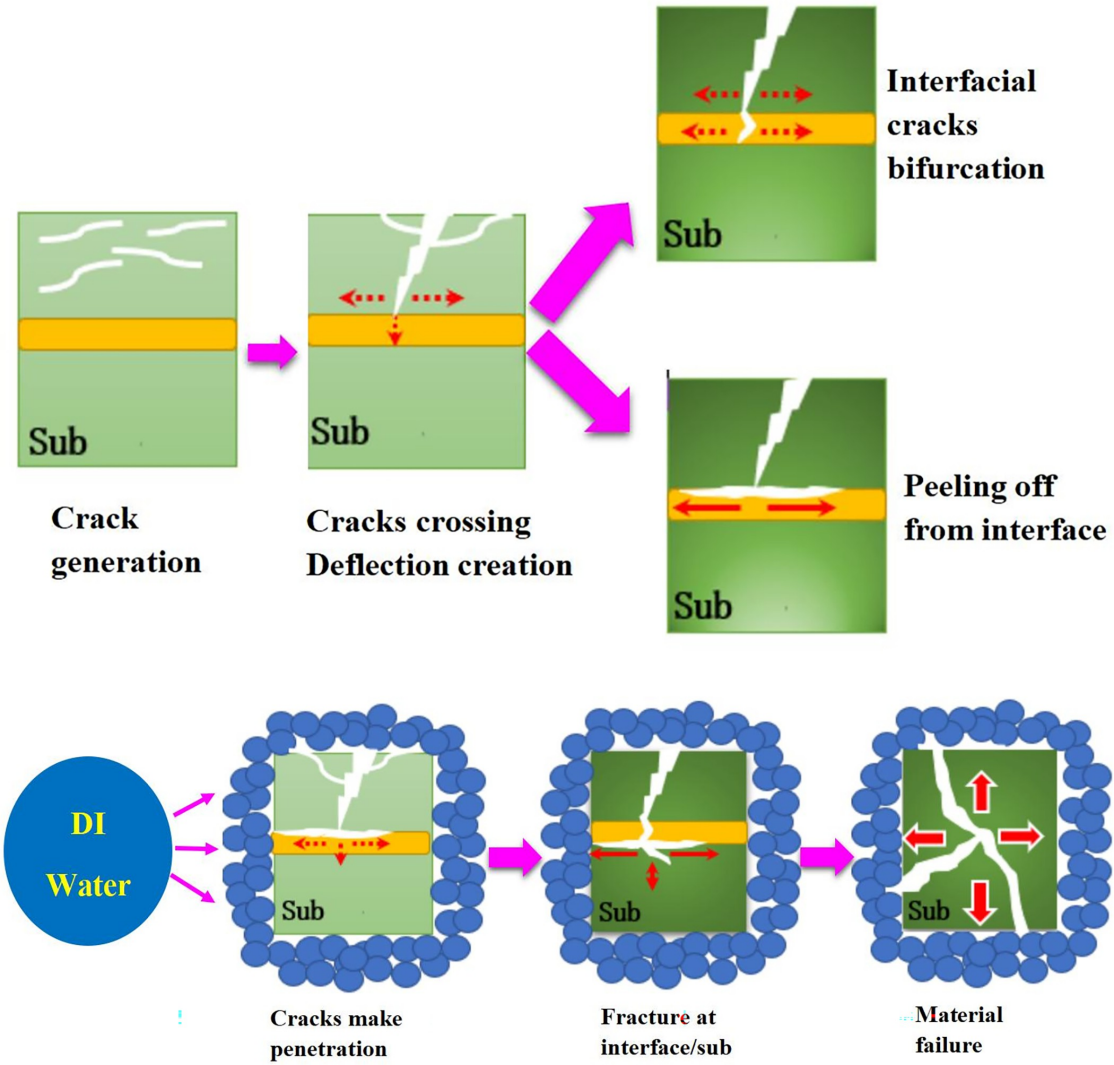
Schematic showing the crack extension formed by 60CO γ irradiation of PMMA. (a) in air medium, (b) in DI water medium.

Fig 18 shows the schematic of crack extension formed by ^60^CO γ irradiation aging of PMMA. In this experiment, we found that the impregnating medium was simultaneously involved in the surface aging change of the material by accelerating the aging of PMMA via ^60^CO irradiation. From the light micrographs, we determined that with the aging process, the surface color of the material changed (Figs 3 and 4), its yellowness also changed significantly (Fig 9), and the cracks and internal structure of the material changed characteristically (e.g., feather-like and snowflake-like in Fig 3). Erosion refers to the phenomenon that the surface of the object is destroyed by chemical reaction, which can lead to the destruction and aging of various materials such as metal, plastic, pottery, glass, etc. Erosion has a serious negative impact on materials: It can lead to changes in the mechanical properties, physical properties, and chemical properties of metal parts, seriously affecting their function, but also lead to changes in appearance, and reduce their service life. The roughness of the sample surface, such as crack, the impurity composition of the sample surface, and the distribution of the elements on the sample surface will all influence the corrosion of the material. A comparison between air and DI water media was also performed, and we found that the addition of DI water did not allow the yellowness to develop as it did in air medium (Fig 10). The light and electron microscopy studies showed that the crack’s origin and development were through competition and selection. That is, the cracks caused further structural and property changes in the material by changing different orientations. We determined that cracks initially arise without a clear pattern. However, a coarse primary crack always appears, and the intersection of primary and secondary cracks frequently leads to vertical cracks. Vertical cracks are first formed on the material surface, and due to the change in the tensile, viscous properties of the material caused by irradiation (Figs 11 and 12), the cracks do not penetrate the material directly but instead manifest themselves by lingering at the middle interface of the material and progressing further down the material. Crack extension has two potential paths along the interface and vertical directions. The deflection of stress leads to the further occurrence of material aging. When irradiation dose reached 1000 kGy, aging reached its extreme in this experiment. In air medium, the cracking of the material occurred only at the surface and the intermediate interface and did not cause the failure of the material. In DI water medium, cracking extended to the extent that cracks entered the intermediate interface to the underside, and eventually, cracks took shape in all directions inside the material. The internal stress of the material changed, leading to the failure of the material. Under the same aging conditions, the degree of destruction of the appearance, morphology, and spatial structure of PMMA under 50 kGy irradiation dose was considerably smaller than that of the PMMA samples under irradiation doses of 500 kGy and 1000 kGy. The appearance and spatial structure damage of PMMA in air medium are significantly lighter than in DI water medium. Crack extension in the vertical direction is observed, and no regular extension of internal crack growth is evident. They are combined with many literature reports because internal stresses cause cracks. When the irradiation medium is water, water will cause a sudden drop in temperature on the sample surface as irradiation progresses. The long-term change between hot and cold will lead to internal stress in the material, leading to increased cracks on the surface. Under air conditions, PMMA exhibits good anti-aging performance, while the addition of DI water significantly reduces PMMA’s anti-aging performance.

## 5. Conclusions

In this study, we found that the addition of medium and the increase of irradiation dose simultaneously affect the cracking of PMMA material. the structure of PMMA is changed, cracks are generated, and the toughness of the samples is reduced. The crack initiation phase and the crack extension phase exhibit different characteristics. Primary cracks are always present initially. Conversely, secondary cracks lead to different stress directions depending on the direction in which they occur. Cracks compete in the irradiated PMMA material, exhibiting crack extension, branching and crossover, and eventually leading to delamination and failure.

The main change in irradiated PMMA is the cleavage. After cleavage, the polymer backbone breaks under high-energy radiation and the polymer molecular chains decrease with increasing absorbed dose, even becoming monomeric molecules. Meanwhile, the irradiation stability is mainly affected by the energy transfer within the polymer, which is related to the spatial effect of the molecules’ materials are stimulated by extreme irradiation doses. Its yellowness increases, light transmission decreases, surface wettability decreases, viscosity increases, and optical properties decrease, further confirming that the main mechanism affecting PMMA under irradiation is the degradation of the material. We found that the thermal stability properties of the materials did not change significantly during the increase of irradiation dose, and the DSC curves were aligned consistently, but the glass transition temperature decreased with the increase of irradiation dose. Combined with the GPC results, 60Co-γ irradiation leads to a decrease in the relative molecular mass of PMMA, but no new moieties are generated. This indicates that the high dose irradiation produced little effect on the molecular composition of the PMMA material.

PMMA is more conducive to retarding cracking after irradiation in air medium, i.e., it is more ductile in air medium than in deionized water medium. By comparing the changes in yellowness, the addition of deionized water served to inhibit the yellowing of the material under the same irradiation conditions, but significantly enhanced the adhesion properties after aging. After irradiation, the hardness of PMM material decreased and was significantly higher than that of deionized water in air. The involvement of deionized water in the cooling process of the material also led to the deterioration of its thermal stability. However, the addition of DI water reduced the glass transition temperature of PMMA after irradiation to some extent, which may be due to the fact that DI water hindered the crystallization of PMMA, which led to the reduction of grain size and lower glass transition temperature than air medium.
